# *In vitro Candida albicans* biofilm formation on different titanium surface topographies

**DOI:** 10.1080/26415275.2020.1829489

**Published:** 2020-10-09

**Authors:** Mathieu Mouhat, Robert Moorehead, Craig Murdoch

**Affiliations:** a Department for Clinical Dentistry, Faculty of Health Sciences, The Arctic University of Norway (UiT), Tromsø, Norway; b School of Clinical Dentistry, The University of Sheffield, Sheffield, UK; c The Henry Royce Institute, The University of Sheffield, Sheffield, UK

**Keywords:** Dental implants, *Candida albicans*, titanium, surface roughness, surface tension

## Abstract

**Objectives:**

To investigate if differences in titanium implant surface topography influence *Candida albicans* biofilm formation.

**Materials and Methods:**

Titanium discs were prepared and characterized using a profilometer: Group A (*R*_a_ 0.15 µm, smooth), Group B (*R*_a_ 0.64 µm, minimally rough) and Group C (*R*_a_ 1.3 µm, moderately rough). Contact angle and surface free energy (SFE) were determined for each group. Non-preconditioned titanium discs were incubated with *C. albicans* for 24 h. In additional experiments, the titanium discs were initially coated with human saliva, bovine serum albumin or phosphate-buffered saline for 2 h before incubation with *C. albicans* for 24 h. The amount of fungal biofilm formation was quantified using a colorimetric assay.

**Results:**

*C. albicans* biofilm formation was significantly lower (*p* < 0.05) on the minimally rough titanium surface compared to smooth and moderately rough surfaces. The titanium surface displaying the lowest SFE (Group B) was associated with significantly lower (*p* < 0.05) *C. albicans* biofilm formation than the other two groups. Salivary coating resulted in greater adherence of *C. albicans* with increased surface roughness.

**Conclusions:**

The minimally rough titanium discs displayed lowest SFE compared to smooth and moderately rough surfaces and showed the least *C. albicans* biofilm formation. This study demonstrated that *C. albicans* biofilm formation increased in a SFE-dependent manner. These findings suggest that SFE might be a more explanatory factor for *C. albicans* biofilm formation on titanium surfaces than roughness. The presence of a pellicle coating may negate the impact of SFE on *C. albicans* biofilm formation on titanium surfaces.

## Introduction

Dental implants made of titanium have been in use for nearly 50 years and are considered an appropriate treatment to manage tooth loss [1] with a cumulative survival rate ranging from >95% over a 7-year period to 85% over 10 years [2–4]. Although traditional dentures may be a good alternative to replace missing teeth, dental implants have several advantages over removable prostheses made from metal, ceramics or polymers-based frameworks. These removable prostheses often pose problems for patients such as metallic taste, risk of hypersensitivity, cytotoxicity, lack of biocompatibility, osteolysis of abutment teeth and increased microbial biofilm formation [5].

The oral microbiome is reported to contain over 700 species and includes Gram-positive bacteria with genus such as *Actinomyces, Bifidobacterium, Corynebacterium, Eubacterium, Lactobacillus, Propionibacterium, Pseudoramibacter, Rothia*, and Gram-negative organisms with genus *Campylobacter, Capnocytophaga, Desulfobacter, Desulfovibrio, Eikenella, Fusobacterium, Hemophilus, Leptotrichia, Prevotella, Selemonas, Simonsiella, Treponema, Wolinella.* Non-bacterial species such as protozoa, viruses and fungi (mainly *Candida, Cladosporium, Aureobasidium, Saccharomycetales, Aspergillus, Fusarium and Cryptococcus*) are also present [6]. Some of these organisms are able to attach to the oral mucosa, tooth enamel or any inert surface placed in the oral cavity [7,8]. This includes implant surfaces where the microbial community can provoke the development of periodontal and peri-implant diseases [9,10].

*Candida albicans* is the most prevalent fungal organism found in the oral cavity being detected in approximately 50% of the population at any given time [11]. For healthy individuals, *C. albicans* is a commensal organism and its presence in the oral cavity remains non-pathogenic [12]. However, *C. albicans* is often termed an opportunistic organism and if levels of colonisation to a surface are not controlled (e.g. in immunocompromised individuals or those receiving high antibiotic therapy), it may cause local tissue damage as observed in pseudomembranous candidiasis and denture stomatitis [13]. The presence of *Candida* species, in particular *C. albicans* in peri-implant lesions has been shown by several groups [14–16]. *Candida albicans* biofilm formation on implant surfaces leads to an overall elevated microbial burden in these niches, increasing the likelihood of *Candida*-associated peri-implantitis that has been implicated, in some studies, in contributing to implant failure [17,18].

Along with host salivary factors and glycoprotein conditioning of the implant surface following implantation; host immunodeficiency, co-aggregation to commensal oral bacteria, the surface physical and chemical properties of materials such as roughness, hydrophilicity, electrostatic forces and surface free energy (SFE) all significantly influence *C. albicans* biofilm formation [19]. On one hand, an appropriate level of roughness is critical for effective osseointegration of a titanium implant with an optimal roughness suggested to be in the region of *R*_a_ of 1.0 − 2.0 µm [20]. On the other, several studies have shown that implant surface roughness is important for biofilm formation with biofilm thickness increasing with increasing surface roughness [21,22]. Commercially available titanium implants have large differences in their surfaces microstructure and topography depending on the manufacturing technique with a range of surface roughness (*R*_a_) between 0.3 µm to over 2 µm [23,24]. Bacterial biofilm formation on titanium surfaces has been previously investigated [25–29] and these studies concluded that bacteria leakage occurred along the implant-abutment interface where implant surface roughness influenced initial bacterial adhesion.

Few studies have examined *Candida* biofilm formation on implants and of those that have provided conflicting data. For example, Quirynen et al. [30] showed that increased titanium surface roughness was associated with greater biofilm formation and Tsang et al. [31] showed that it was associated with decreased antifungal susceptibility, whilst others found that SFE rather than surface roughness was associated with biofilm formation [32,33]. Conflicting evidence concerning the influence of SFE on fungal biofilm formation also exists [32,34–36]. Beside surface roughness, fungal biofilm formation may also be influenced by the presence of a salivary pellicle that coats the titanium implant [21,37,38] with both increased and decreased fungal formation being reported [32,33].

Therefore, the aims of this study were to evaluate the effect of surface roughness, SFE and the influence of the salivary pellicle on *C. albicans* biofilm formation on clinically relevant titanium implant surfaces.

## Materials and methods

### Sample preparation

A commercially pure titanium tube (99.6% pure Titanium, Goodfellow, Coraopolis, USA) was cut into 27 discs of 10 mm diameter and 1.2 mm thickness with a precision cutting machine (IsoMet 1000, Buehler, Lake Bluff, USA) using an abrasive cut-off wheel (Type T5, MetPrep, Coventry, UK). Grinding was performed sequentially by one trained operator applying a constant pressure. All 27 discs were ground manually on one side with grit size P60 metallographic abrasive paper (P60, MetPrep, Coventry, UK) on a grinder-polisher (Metaserv 5, Buehler, Lake Bluff, USA) for one minute. Nine discs were removed and all the remaining discs were ground for one minute with P120 metallographic abrasive paper. Nine discs were again removed and the remaining nine discs ground for one minute with P600 metallographic abrasive paper.

### Surface characterisation

The surface roughness (*R*_a_) of the discs after grinding with 60, 120 and 600 metallographic abrasive paper, respectively (*n* = 9 in each group) were quantified with a profilometer using a 0.2 µm diamond tip (TR200, Time Group, UK) at three different locations on each disc. The titanium discs were then divided in three groups (Group A smooth, Group B minimally rough and Group C moderately rough) according to the classification proposed by Wennerberg & Albrektsson (2009) [20]. The sessile drop method was used to measure the contact angle of ultrapure water or diiodomethane (Fischer Scientific, Leicester, UK) each with different hydrophobicity. A 5-µl droplet with known polar and dispersive fractions of the surface tension was placed on the surface of titanium discs and then SFE was calculated from contact angle measurements (i.e. the measurements of the angles made by the intersection of the liquid/solid interface) using a drop shape analyser (DSA100, Krüss, Hamburg, Germany). In total, five droplets of each liquid for each sample were examined. The SFE as well as its dispersion and polar components were subsequently calculated according to the Owens, Wendt, Rabel and Kaelble equation [39] using ADVANCE software (Krüss, Hamburg, Germany). One titanium disc from each roughness group was selected for imaging using scanning electron microscopy (SEM) (Vega3, Tescan, Brno, Czech Republic).

### In vitro Candida albicans growth

*C. albicans* strain CAF2-1 was used in this study. CAF2-1 (stored at −80 °C) was defrosted and grown on 1% yeast, 2% peptone and 2% dextrose (YPD, all from ThermoFisher Scientific Oxoid, Basingstoke, UK) agar plates in an incubator at 37 °C for 24 h. Then, a loop of *C. albicans* was placed in 20 ml YPD broth and cultured at 25 °C in an orbital shaker at 100 revolutions per min (rpm) for 24 h. The *C. albicans* suspension was centrifuged at 3000 rpm for 5 min, supernatant discarded and the *C. albicans* pellet washed with 20 ml phosphate-buffered saline (PBS, Sigma-Aldrich, Poole, UK) solution twice. An aliquot of *C. albicans* was removed for counting using a haemocytometer (improved Neubauer with 0.1 mm depth and 1/400 mm^2^).

### In vitro Candida albicans biofilm formation on non-pre-conditioned titanium disc surfaces

The XTT biofilm assay relies on the metabolism of the XTT dye to form a colour product by viable *C. albicans*. Although XTT measures cellular metabolism the optical density (OD) of the colour product is directionally proportional to the number of viable Candida [40]. To empirically determine the optimal level of *C. albicans* required to produce maximal biofilm formation, increasing quantities of *C. albicans* were prepared in Roswell Park Memorial Institute-1640 media (RPMI, Sigma-Aldrich, Poole, UK) and aliquoted into each well of a tissue culture-treated 48-well plate (Sigma®cell culture plate, Sigma-Aldrich, Poole, UK) ranging from 6.25 × 10^4^ to 4 × 10^6^ CFU/ml and incubated for 24 h to form a biofilm. RPMI was removed and biofilm gently washed with PBS to remove non-adherent *C. albicans*. 300 μl of XTT (2,3-bis2-methoxy-4-nitro-5-sulfophenyl-2H-tetrazolium-5-carboxyanilide, Sigma-Aldrich, Poole, UK) was then added in each well and incubated for 20 min at 37 °C. 100 µl of the XTT solution from each well were transferred to corresponding wells of a 96-well cell plate and the OD measured by spectrophotometer (Infinite 200 PRO, Tecan, Männedorf, Switzerland) at 450 nm with a correction reading taken at 650 nm. The titanium discs previously submitted to the physicochemical analyses were reused by decontaminated with 70% ethanol then rinsing in sterile PBS prior each experiment. Discs were placed in a 48-well plate and incubated with 300 µl *C. albicans* suspension (3 × 10^5^ CFU/ml) at 37 °C for 24 h. Biofilm growth of the same density of *C. albicans* on tissue culture-treated plastic having the same surface area as the titanium discs was used as a positive control in all experiments. One titanium disc from each roughness group was selected for imaging using SEM. The wells were washed gently twice with excess PBS, 300 µl of TOX-2 XTT assay reagent (Sigma-Aldrich, Poole, UK) added and the plate incubated at 37 °C for 20 min. The XTT solution was also added in three empty wells to calculate a background OD. 100 µl of the XTT solution from each well was transferred to a well of a 96-well cell plate and the OD measured at 450 nm as previously described. Each individual experiment was performed in triplicate for each surface roughness. The independent experiments were performed in three different days using different titanium discs and *C.albicans* culture.

### Scanning electron microscopy

SEM analysis was performed on plain and *C. albicans*-treated non-pre-conditioned titanium discs for morphological validation. One plain titanium disc was selected from each roughness group (*n* = 3). Similarly one *C. albicans*-treated non-pre-conditioned disc from each roughness group was selected (*n* = 3). Prior the SEM analysis, *C. albicans* were fixed with a 2.5% glutaraldehyde solution (Agar Scientific, Stansted, UK) in 0.1 M sodium phosphate. The specimens were washed twice (with 10 min intervals) in 0.1 M phosphate buffer then dehydration performed with a graded series of ethanol (75% ethanol for 15 min; 95% ethanol for 15 min; 100% ethanol for 15 min and 100% ethanol dried over anhydrous copper sulphate for 15 min). Discs were placed for 30 min in hexamethyldisilazane, air dried overnight and mounted on aluminium stubs, attached with carbon sticky tabs, and sputter coated with gold (S150B, Edwards High Vacuum International, Burgess Hill, UK). Images were acquired at ×500 and ×5000 magnification from different areas of the discs in a high vacuum mode at an accelerated voltage of 12 kilovolt (Kv) and the height of the electrode was approximately 10 mm.

### In vitro salivary pellicle formation on titanium disc surfaces

Whole human saliva was collected into a sterile tube by expectoration from a healthy 33-year-old volunteer without periodontal diseases or active carious lesions and who refrained from eating, drinking and oral hygiene procedures for 8 h prior to collection with written, informed consent (University of Sheffield Research Ethics Committee approval number 003166). Their unstimulated salivary flow rate was 0.3 ml/min. The saliva was first centrifuged at 2500 rpm for 3 min to pellet debris and then sterilized with single-use filters with pore size of 0.45 µm then 0.20 µm (StarLab, Milton Keynes, UK) as previously described [32]. Prior to the *C. albicans* assay, 300 µl of human whole saliva, bovine serum albumin (BSA, Sigma-Aldrich, Poole, UK) (40 µg/ml; Sigma-Aldrich, Poole, UK), or PBS were added to discs and incubated for 2 h at room temperature. After removing the surplus solutions, a thin pellicle coating on each disc was left before investigating the *in vitro C. albicans* biofilm formation on the titanium discs surface.

Supplementary Figure 1 describes the steps of the materials and methods.

### Statistical analyses

Statistical analysis was performed in the statistical package for the social sciences (SPSS) version 25 (IBM corporation, New York) and GraphPad Prism version 8 (GraphPad software, San Diego, USA). Unless otherwise stated data are presented as mean ± standard deviation (SD). Normality of data was determined using D’Agostino & Pearson normality test. Non-parametric data was analysed using Kruskal–Wallis followed by Dunn’s post hoc analysis. Parametric data was analysed by One-way ANOVA followed by Tukey’s *post hoc* analysis.

## Results

### Roughness, SFE and morphology of the titanium disc surfaces

Initially, a dose-response experiment was performed to determine optimal *C. albicans* biofilm formation to tissue culture-treated surfaces (positive control). Biofilm formation to culture plastic was dose-dependent reaching maximal levels at 5 × 10^5^ CFU/ml (Supplementary Figure 2). Therefore, 3 × 10^5^ CFU/ml *C. albicans* was used in subsequent experiments as this provides a high level of biofilm formation whilst being directionally proportional to the OD.

**Figure 1.**
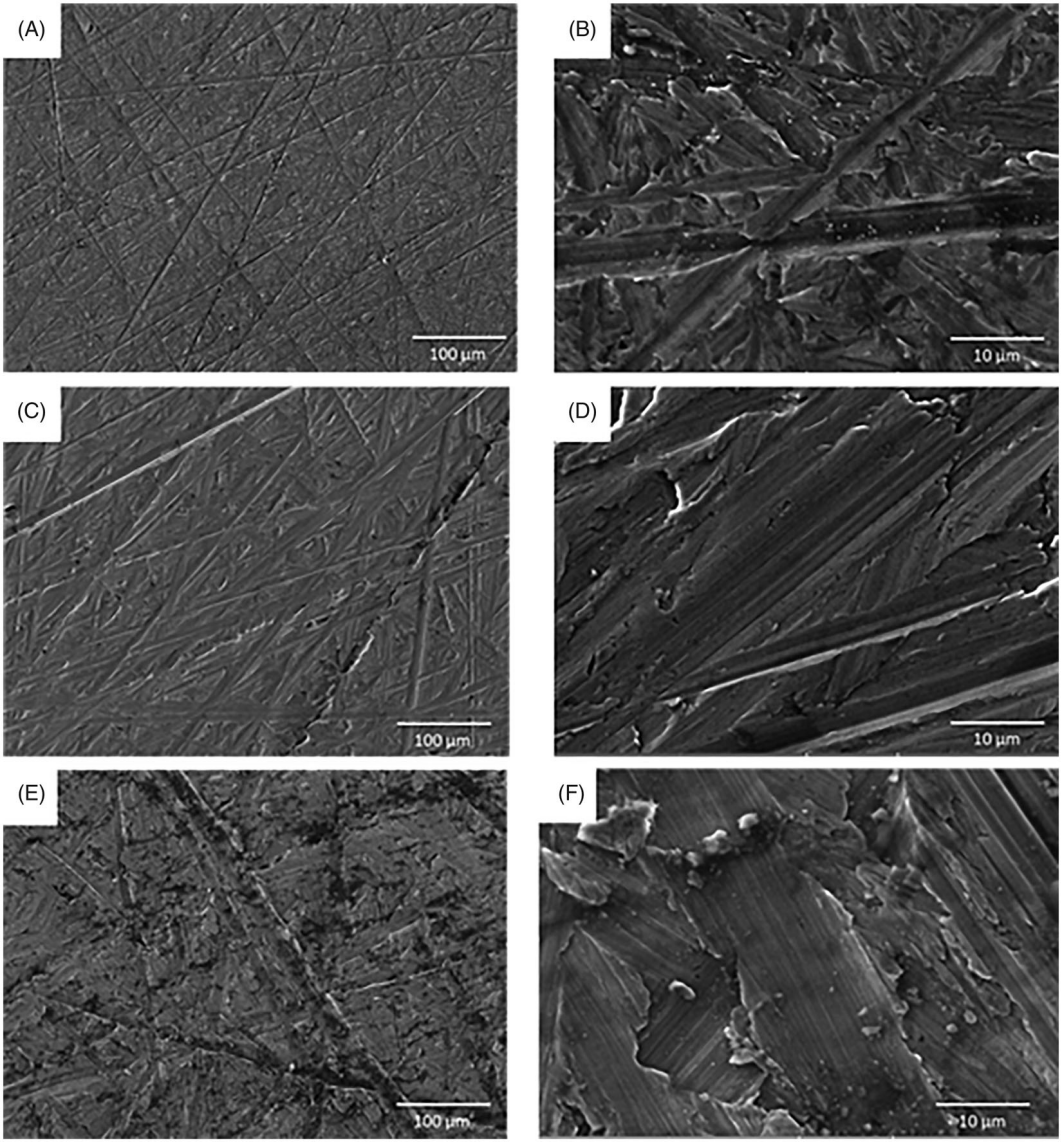
Scanning electron microscope (SEM) micrographs showing A&B) smooth, C&D) minimally rough and E&F) moderately rough titanium discs surface. Scale bar for images in A, C and E = 100 μm, Scale bar for images in B, D and F = 10 μm.

The mean surface roughness of Group A (smooth) was *R*_a_ 0.15 ± 0.02 µm, Group B (minimally rough) R_a_ 0.64 ± 0.08 µm and Group C (moderately rough) *R*_a_ 1.30 ± 0.05 µm (Table 1). The mean contact angle value was the highest for the Group B (86.15° with water droplet and 62.80° with diiodomethane droplet) and therefore it was the most hydrophobic, followed by the Group A (74.47° with water droplet and 55.93° with diiodomethane droplet) and then Group C (62.01° with water droplet and 55.39° with diiodomethane droplet) being the least hydrophobic (Table 1). There was significant difference in the mean values of surface roughness between all three groups (*p* < 0.001) and in contact angle between Group B and C (*p* < 0.001; Table 1). Group C displayed the significantly greater SFE to Group B (*p* < 0.01) but not Group A (Table 1). SEM images corroborated the titanium surface roughness measurement data, showing disc surfaces with alternated flat and grooved surfaces that increase in width and depth from smooth (Figure 1(A,B)) to minimally rough (Figure 1(C,D)) and to moderately rough (Figure 1(E,F)).

**Table 1.** Surface roughness, contact angle and surface free energy values (mean ± SD) of the three titanium discs groups.

		Group A (smooth)	Group B (minimally rough)	Group C (moderately rough)
Roughness R_a_ (µm)		0.15 ± 0.02^a^	0.64 ± 0.08^a^	1.30 ± 0.05^a^
Contact angle (°)	Water	74.5 ± 10.2	86.2 ± 10.0^b^	62.0 ± 10.0^b^
	Diiodomethane	55.9 ± 4.2	62.8 ± 7.1	55.4 ± 5.3
Surface free energy (mJ/m^2^)		39.4 ± 6.3	31.8 ± 6.5^c^	46.1 ± 7.2^c^
[Dispersion component/polar component]		[30.91/8.45]	[27.04/4.67]	[31.19/14.88]

The letters a, b and c indicate statistically significant differences of at least *p* < 0.01 between these groups using one-way ANOVA followed by Tukey’s post-hoc multiple comparisons test..

### Candida albicans biofilm formation on non-pre-conditioned titanium discs

**Figure 2.**
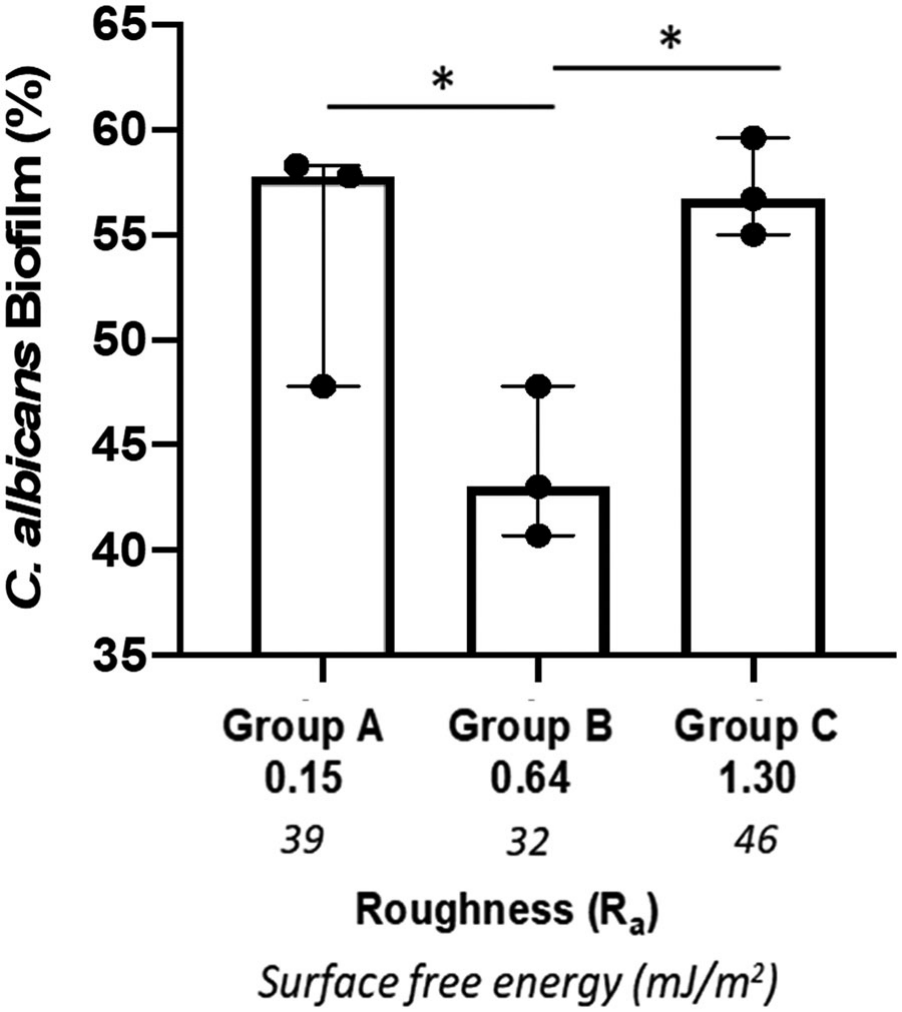
*C. albicans* biofilm formation on titanium discs. The amount of biofilm formation (%) on titanium discs of increasing surface free energy (SFE) and with different roughness (*R_a_ µm*) was calculated using an XTT assay. Data displayed are median ± interquartile range of 3 independent experiments repeated on different occasions using different titanium discs and *C.albicans* culture each performed in triplicate. *p < 0.05 using Kruskal-Wallis followed by Dunn's *post hoc* multiple comparison statistical test.

**Figure 3.**
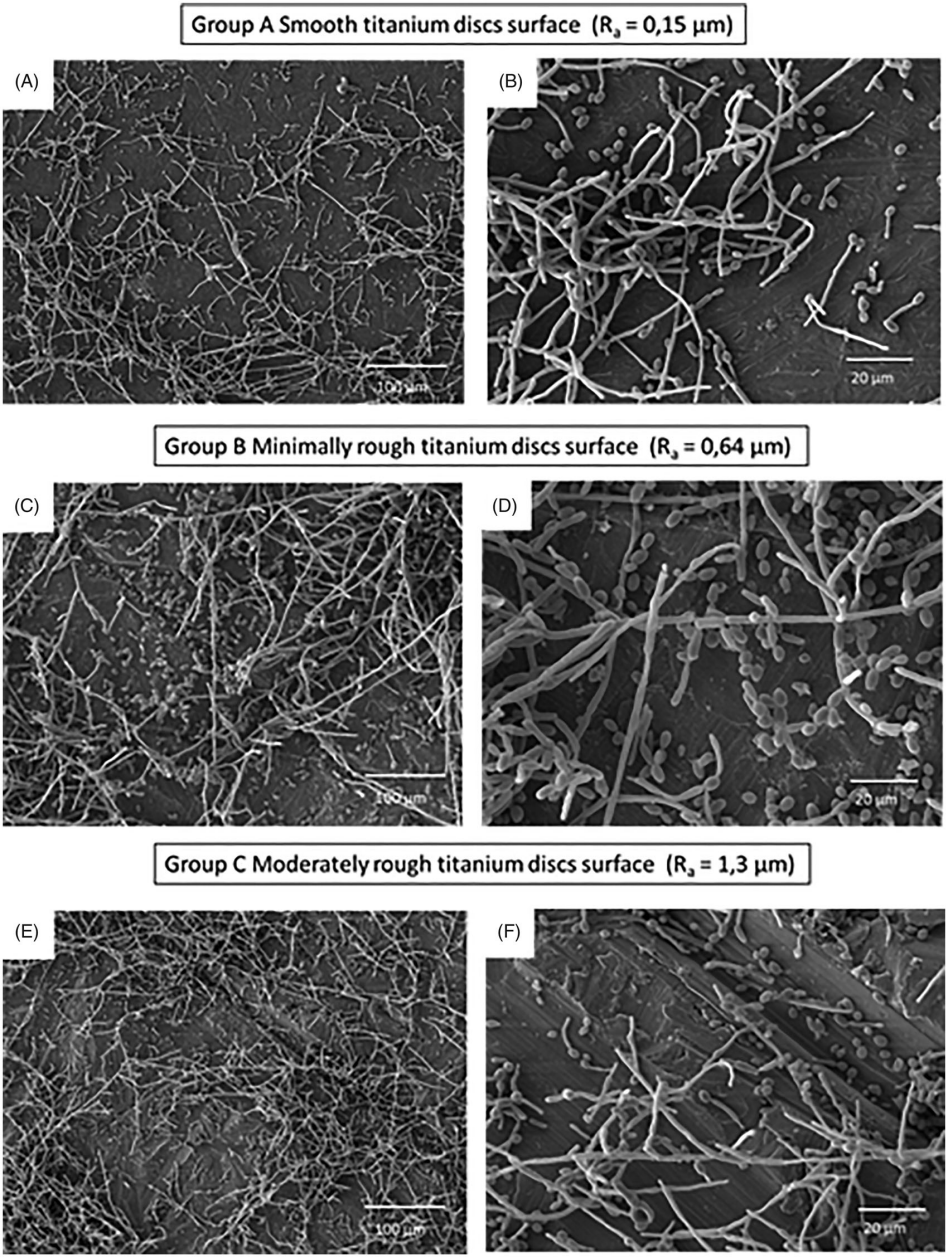
Scanning electron microscope (SEM) micrographs of the three different titanium discs surfaces showing *C. albicans* biofilm formation. Scale bar is 100 µm (A,C,E) and 20 µm (B,D,F).

*Candida albicans* were incubated for 24 h on titanium discs or on tissue culture plastic of the same diameter as a positive control. On tissue culture plastic, biofilm formation was extensive and was set at 100%, allowing comparison of biofilm formation on the increasingly rough surfaces. *C. albicans* biofilm formation to the titanium discs was significantly lower (*p* < 0.001) than to tissue culture plastic irrespective of surface roughness. However, when analysed in the order of increasing roughness (*R*_a_) Group C (*R*_a_ 1.3, moderately rough) and Group A (*R*_a_ 0.15, smooth) displayed similar levels of biofilm formation on the titanium discs with levels of 57.1 ± 2.3% and 54.3 ± 6.5%, respectively. In contrast, biofilm formation on the minimally rough (*R*_a_ 0.64) titanium disc surface was significantly lower (43.8 ± 3.6%) when compared to that on both smooth (*p* < 0.05) and moderately rough (*p* < 0.05) discs (Figure 2). Subsequently, *C. albicans* biofilm formation in relation to differences in SFE was analysed. Here it has been found that biofilm formation increased in an SFE-dependent manner, with Group B (lowest SFE, minimally rough) displaying significantly (*p* < 0.05) lower biofilm levels than Group A (smooth, moderate SFE) and Group C (moderately rough, highest SFE) (Figure 2). Of note, Group C discs that displayed the roughest surface and highest SFE also exhibited most *C. albicans* biofilm formation (Figure 2). SEM images of *C. albicans* attached to titanium discs surface showed the presence of both yeast blastospores and hyphae, with the hyphal form being most prominent (Figure 3). For the moderately rough titanium discs surface (Group C), *C. albicans* growth tracked along the grooves and irregularities of the surface in many instances (Figure 3(E,F)), while for the smooth and minimally rough titanium discs surface (Group A and B) this was less obvious. Moreover, *C. albicans* biofilm were observed mainly in clusters rather than homogeneously spread across the surface (Figure 3).

### Influence of a salivary pellicle on Candida albicans biofilm formation

**Figure 4.**
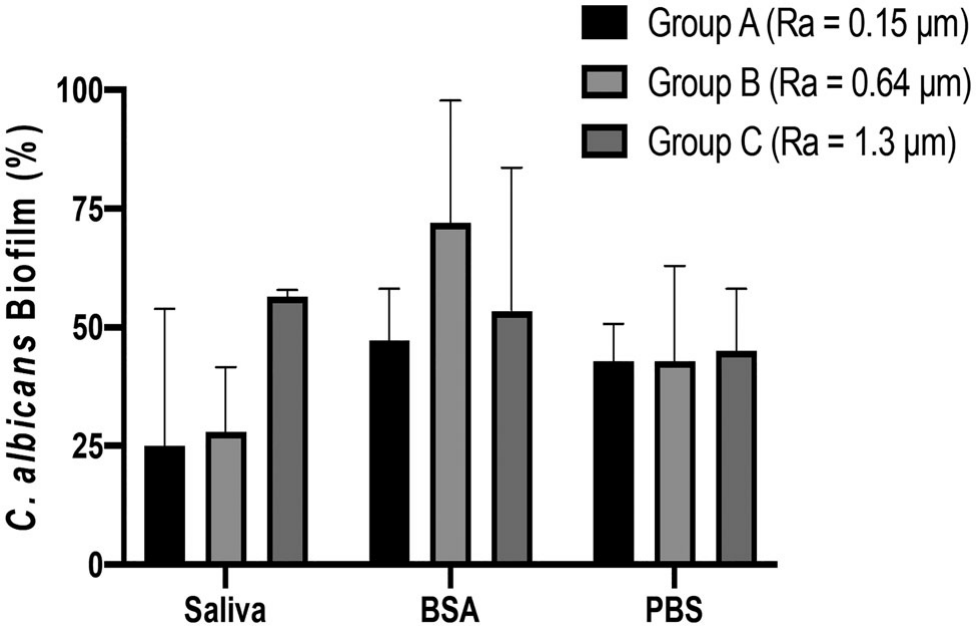
Influence of salivary pellicle on *C. albicans* biofilm formation. Titanium discs of different roughness were pre-coated with PBS, BSA or human saliva and incubated with *C. albicans* for 24 h, Amount of biofilm formation (%) was calculated using an XTT assay. Data displayed are median ± 95% CI of 3 independent experiments. Data were analysed by Kruskal-Wallis followed by Dunn’s post-hoc analysis for multiple comparisons.

Addition of a salivary pellicle did not significantly alter *C. albicans* biofilm formation on smooth titanium discs (*R*_a_ 0.15) compared to BSA or PBS treated discs (Figure 4). On minimally rough discs (*R*_a_ 0.64) biofilm formation of *C. albicans* was increased upon treatment with BSA compared to PBS controls whilst addition of a salivary pellicle resulted in variable biofilm formation (Figure 4). For the moderately rough group (*R*_a_ 1.3) coating with a salivary pellicle resulted in markedly increased *C. albicans* biofilm formation compared to that observed with BSA or PBS, although these differences were not statistically significant (Figure 4). Human saliva pellicle and titanium topography were directly associated; with increased biofilm formation dependent on surface roughness (Figure 4). No statistically significant difference in *C. albicans* biofilm formation was found between the three groups of titanium discs coated with PBS (Figure 4). *C. albicans* biofilm formation was lower for the PBS-coated titanium discs surfaces compared to the uncoated discs surfaces within each roughness group (Figures 2 and 4).

## Discussion

The first step in *C. albicans* infection is its adhesion on a surface leading to biofilm formation [8,41] and therefore surface properties, such as roughness, hydrophobicity and SFE may have a major influence on fungal colonisation [19,35]. While *C*. *albicans* colonisation on polymeric denture materials and its association with denture stomatitis have been extensively studied [42], such investigations on titanium surfaces are relatively scarce in the literature. The present study demonstrated that a minimally rough titanium surface displayed significantly lower *C. albicans* biofilm formation compared to both smooth and moderately rough surfaces. The minimally rough titanium discs had the lowest SFE compared to smooth and moderately rough surfaces. *C. albicans* biofilm formation increased in a SFE-dependent manner suggesting that SFE might be a more explanatory factor for *C.albicans* biofilm formation on titanium surfaces than roughness. The addition of a salivary pellicle to the titanium surface resulted a linear relationship between surface roughness and *C. albicans* biofilm formation, though there was no statistically significant difference between the groups This results may suggest that the addition of a pellicle is likely to nullify the impact of SFE on *C. albicans* biofilm formation on titanium surfaces.

### Candida albicans biofilm formation on non-pre-conditioned titanium discs

The results of this study are in line with several previous studies that have demonstrated a relationship between surface roughness and *C. albicans* colonisation; the rougher titanium surfaces resulted in increased *C. albicans* biofilm formation, although the differences between group A (the smoothest surface (smooth)) and group C (the roughest surface (moderately rough)) did not reach statistical significance [31,43]. Interestingly, in the present study, the titanium discs in Group A (smooth) and those in Group C (moderately rough) displayed significantly more *C. albicans* biofilm formation compared to average rough surface of Group B (minimally rough). These results suggest that similar to osseointegration, where optimal roughness is preferable for improved implant incorporation into bone [20,44], an optimal roughness, which is neither too low nor too high, might be beneficial for minimal *C. albicans* biofilm formation. Of note, that these results were generated on non-pre-conditioned titanium discs. An increase in surface roughness provides shelter within surface irregularities [30]. However, Group A with a smooth titanium surface group, displayed increased *C. albicans* biofilm formation compared to Group B, which displayed a minimally rough surface. Group A surfaces exhibited higher SFE than Group B, suggesting that the influence of SFE may be more explanatory than the influence of roughness for *C. albicans* biofilm formation on titanium surface.

This study showed a linear relation between SFE and *C. albicans* biofilm formation on titanium substrata where the lowest *C. albicans* biofilm formation was found on Group B, which displayed the lowest SFE. This result is in line with several other studies that have shown decreased *C. albicans* biofilm formation with lower SFE on resin materials [35,36] and on titanium coated with silane primer that decreased their SFE [45]. Some evidence suggests that hydrophilic surface, which usually yields high SFE, promotes increased wettability and enhances the interaction between the implant surface and the biologic environment [46,47] including *C. albicans* [48]. In this study, Group C was the most hydrophilic, displayed the highest SFE and resulted in a statistically significantly higher *C. albicans* biofilm formation compared to Group B. Contact angle measurements are commonly employed to define the hydrophilicity/hydrophobicity of a surface. The surface is considered hydrophilic with a contact angle which is >90° and hydrophilic when a contact angle is < 90° using static water [49]. In this study, the mean contact angle value was the highest for Group B (86.2°) and so this group was considered as the most hydrophobic being very close to, but not reaching, the 90° cut-off point. Some other evidence suggests that hydrophobicity of a material positively correlates with *C. albicans* biofilm formation; the cell surface of *C. albicans* is hydrophobic and specific hydrophobic cell wall proteins such as Csh1p have been shown to play a role in mediating attachment to both host or fabricated surfaces by non-covalent interactions [50,51]. However, it is argued that attachment of *C. albicans* to plastic surfaces is influenced by cell surface hydrophobicity only if the nature of plastic surface is hydrophobic [50]. The measured contact angle on the titanium surfaces in this study showed that no group was ‘true’ hydrophobic (a contact angle < 90°) suggesting the same biofilm formation behaviour on a titanium surface as on a plastic surface, i.e. the cell surface hydrophobicity plays a role only when titanium surface is hydrophobic. Therefore, the cell surface hydrophobicity might not be the main explanatory factor for *C. albicans* biofilm formation on the titanium discs tested. The lowest *C. albicans* biofilm formation on the most hydrophobic surface observed in this study may be explained by *C. albicans* binding to titanium discs occurring through a specific receptor-mediated interaction rather than hydrophobicity. It has been suggested that Agglutinin-like sequence protein family (Flo and Epa adhesin) are important for metal binding [52]. A recent study concluded that *C. albicans* biofilm formation was not significantly different on titanium implant material with changed SFE induced by aging process [53]. Conversely, Bürgers et al. [32] found that sand-blasted titanium, having a very high SFE (69.8 mJ m^−2^), exhibited low fungal biofilm formation, although this data may be explained by experimental differences whereby *C. albicans* colonisation was measured after only 2.5 h compared to 24 h in this study.

### Influence of a salivary pellicle on Candida albicans biofilm formation

There is conflicting data in the literature as to whether the presence of a salivary pellicle enhances or inhibits *C. albicans* biofilm formation [54,55]. In this study, the addition of a salivary pellicle to the titanium surface resulted a linear relationship between surface roughness and biofilm formation. Moreover, non-pre-conditioned minimally rough titanium disc surface that was observed to have the lowest *C. albicans* biofilm formation, when coated with BSA appeared to have a higher biofilm formation compared to the BSA coated smooth and moderately rough surfaces. It has been proposed that addition of a salivary pellicle results in the titanium being coated with host proteins to which *C. albicans* can bind [37]. Indeed, Bürgers et al. [32] found that in the presence of saliva, *C. albicans* biofilm formation was increased on a smooth titanium surface (*R*_a_ 0.11 µm) compared to PBS controls. Similar salivary proteins also have an important role in mediating fungal biofilm formation to biomaterials by modulating their adhesion capacity [56], whilst other studies have shown the importance of albumin in microbial adherence [57–59]. The present study found that the addition of a salivary pellicle increased *C. albicans* biofilm formation to titanium compared to BSA coating, but only for the roughest surface (*R*_a_ 1.3 µm), and even then this was not statistically significant. Bürgers et al. [32] also found increased levels of *C. albicans* with salivary compared to albumin coating despite different surface roughness. The difference in results may be explained by different incubation times undertaken, whereby Bürgers examined *C. albicans* biofilm formation over 2.5 h whereas in this study it was analysed after 24 h. It is possible that analysis at an early time point is able to pick up differences associated with early *C. albicans* biofilm formation, events that are not evident after 24 h when the fungal growth and hyphal transformation is more advanced. However, it must be noted that in both cases differences in biofilm formation to titanium surfaces with different roughness were not significant when treated with either salivary proteins or BSA. Surprisingly, our data showed that PBS treated titanium discs displayed lower *C. albicans* biofilm formation compared to the non-pre-conditioned discs within each roughness group. Of note, these two experimental settings had different time points and therefore a direct comparison must be taken with caution. PBS coating increases hydrophilicity and might therefore increase SFE comparing to non-pre-conditioned discs, resulting in higher *C. albicans* biofilm formation. This indicates that cellular formation cannot be explained only by SFE. Fungal biofilm formation is a complex process that is affected by many factors including environment, cells characteristics, materials surface characteristics and configuration [60].

### Methodological considerations

The strength of this study is that the titanium discs surface roughnesses (*R*_a_) used in our study reflect the roughness of commercially available implants from several companies [23].

This study only used a well-characterised laboratory strain of *C. albicans* (CAF2-1) to enable comparisons to previous studies and to allow the use of mutant strains in follow-on studies. However, strain-to-strain differences may exist, so future studies should extend to the use of clinical isolates and other types of Candida such as *C. glabrata*, *C. krusei* and *C. auris*. Although providing evidence for *C. albicans* biofilm formation, our data does not take into account the influence of other microorganisms present in the oral cavity. It has been hypothesized that oral yeasts co-aggregate in the presence of pathogenic bacteria, increase the virulence of *C. albicans* and accelerate the inflammatory response, which contributes towards the progression of peri-implantitis [61]. For instance, a recent study showed that the interaction of Streptococci from the mitis group with *C. albicans* upregulated the hypha-associated efg1 gene in *Candida* that may drive hyphal-mediated tissue damage [62]. Further experiments using multi-species biofilm may shed more light onto the influence of surface roughness at the implant site as would the inclusion of gingival crevicular fluid flow and shear stress to create continuous detachment forces and so influence biofilm characteristics [63,64].

In conclusion, non-pre-conditioned minimally rough titanium discs (Group B; R_a_ 0.64 µm) showed the least *C. albicans* biofilm formation compared to smooth (*R*_a_ 0.15 µm) and moderately rough (*R*_a_ 1.3 µm) surfaces. The minimally rough titanium discs also displayed the lowest SFE compared to smooth and moderately rough surfaces. The present study demonstrated that *C. albicans* biofilm formation increased in a SFE-dependent manner. These findings suggest that SFE might be a more explanatory factor for *C. albicans* biofilm formation on titanium surfaces than roughness. The addition of a pellicle may negate the impact of SFE on *C. albicans* biofilm formation on titanium surfaces; further studies with a salivary pellicle and multi-species biofilm are needed to validate these results.

## Supplementary Material

Supplemental Material

Supplemental Material

## Data Availability

The datasets generated during and/or analysed during the current study are available from the corresponding author on reasonable request.
