# Supplementary material for: In vitro Candida albicans biofilm formation on different titanium surface topographies
Source: Biomater Investig Dent. 2020 Oct 9;7(1):146–57. doi: 10.1080/26415275.2020.1829489 (PMC7580804; doi:10.1080/26415275.2020.1829489)
Supplement: Supplemental Material [file IABO_A_1829489_SM6766.pdf]

In vitro Candida albicans biofilm formation on different titanium surface topographies

Supplementary Figures

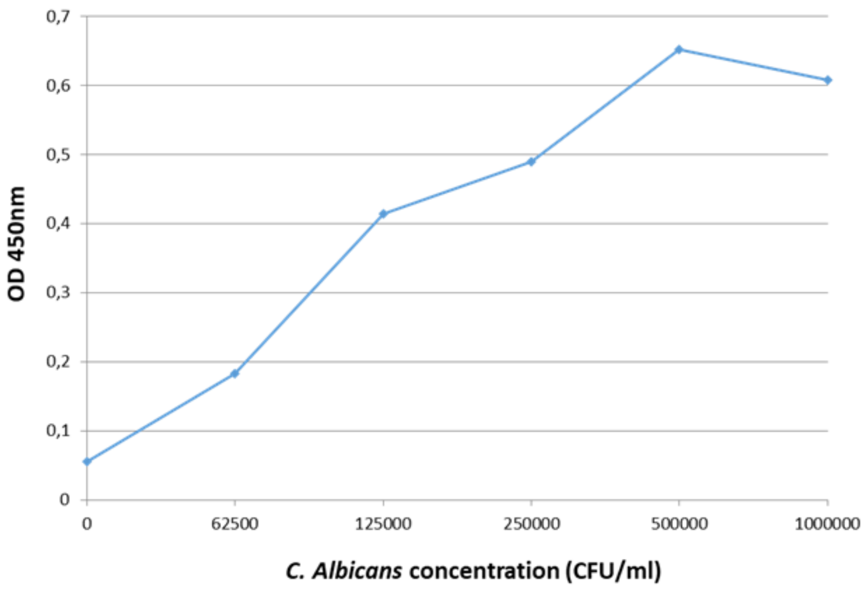

Supplementary Figure 2. XTT formazan signal (measured at 450 nm) produced by *C. albicans* biofilms. Suspensions of *C. albicans* were prepared in RPMI-1640 media ranging from 6.25 x 10^4 to 4 x 10^6 CFU/ml, aliquoted into each well of a tissue culture-treated 48-well plate and incubated for 24 h to form a biofilm. RPMI-1640 was removed and biofilms washed with PBS. XTT (300 µl per well) was added and incubated for 20 minutes at 37°C. 100 µl of the XTT solution from each well was transferred to corresponding wells of a 96-well cell plate and the OD measured by spectrophotometer at 450 nm with a correction reading taken at 650 nm.
