# Supplementary figures and images for: In vitro Candida albicans biofilm formation on different titanium surface topographies
Source: Biomater Investig Dent. 2020 Oct 9;7(1):146–57. doi: 10.1080/26415275.2020.1829489 (PMC7580804; doi:10.1080/26415275.2020.1829489)

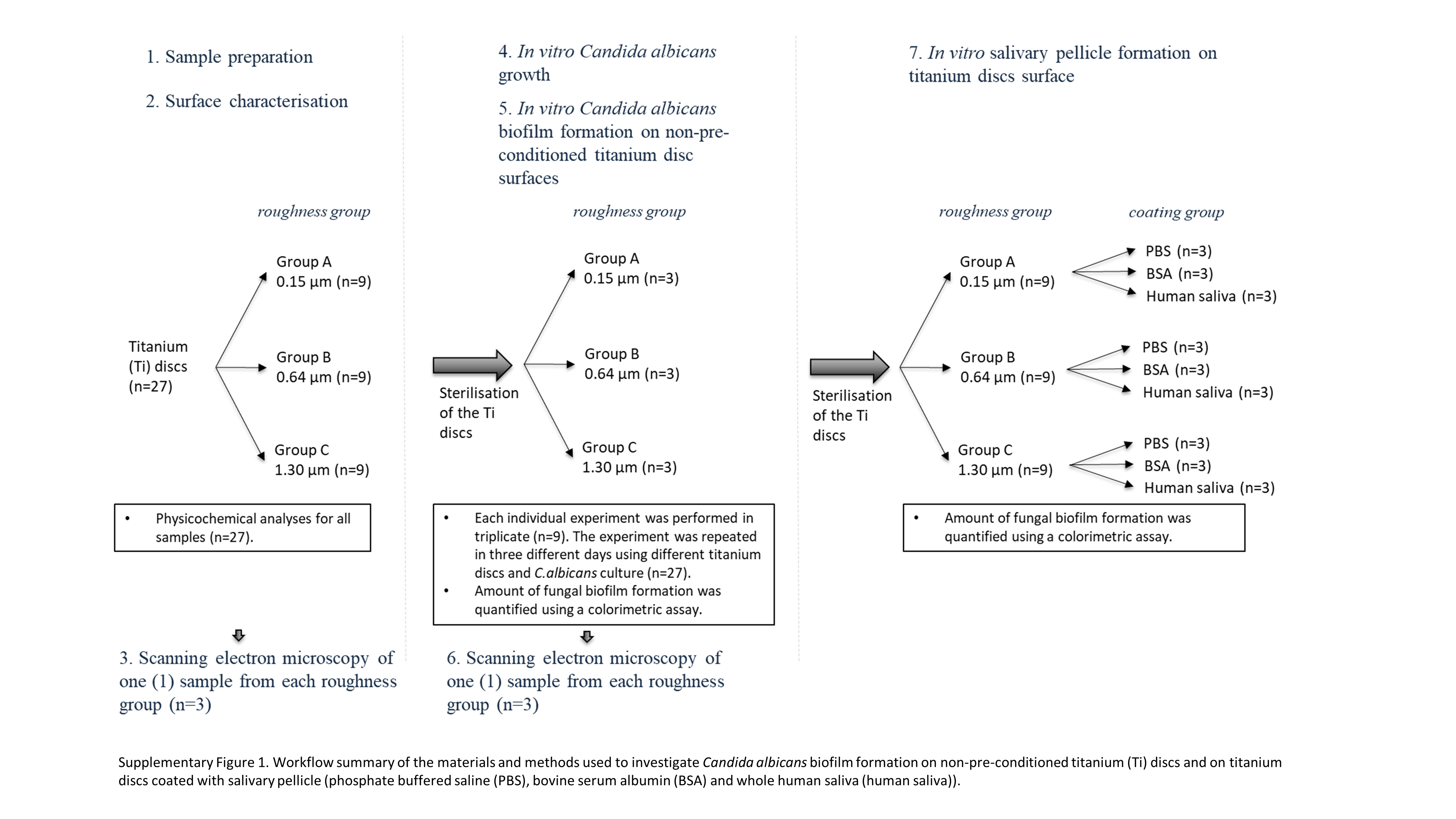

Supplement: Supplemental Material [file IABO_A_1829489_SM6744.tif]
